# Supplementary material for: Associations of multiple trace elements with bipolar disorder in adolescents: A case-control study
Source: PLoS One. 2025 May 6;20(5):e0322958. doi: 10.1371/journal.pone.0322958 (PMC12054906; doi:10.1371/journal.pone.0322958)
Supplement: S1 File — (DOCX) [file pone.0322958.s001.docx]

**Supplementary Materials**

| Fig S1 | The Spearman correlation heatmap of urinary elements’ levels (log) among the overall participants. |
| --- | --- |
| Fig S2 | The elements of depressive phase selected into the multi-element model by LASSO regression |
| Fig S3 | The BKMR models of each element and mixed exposure effects on depression phase. |
| Fig S4 | The elements of manic phase selected into the multi-element model by LASSO regression. |
| Fig S5 | The BKMR models of each element and mixed exposure effects on manic phase. |
| Table S1 | Limits of detection, percentages of samples below detection limits (n=288). |
| Table S2 | Concentrations of elements among study participants (n=288). |
| Table S3 | Concentrations of elements among Depression and Manic (n=144). |
| Table S4 | Odds ratios (ORs) and 95% confidence intervals (95% CIs) for the manic phase versus the depressive phase based on urine element in single-element model (n=144). |
| Table S5 | Multi-element model for adolescent bipolar disorder depression phase associated with multiple elements and characteristics of participants (n=234). |
| Table S6 | Multi-element model for adolescent bipolar disorder manic phase associated with multiple elements and characteristics of participants (n=200). |
| Table S7 | Clinical trials registration of bipolar disorder and trace elements |
| Table S8 | Clinical studies on bipolar disorder and trace elements |
| Table S9 | Basic studies on bipolar disorder and trace elements |

**Table S1.** Limits of detection, percentages of samples below detection limits (n=288).

| Elements | LOD | Total No(%)<LOD |
| --- | --- | --- |
| B | 1.450 | 0 (0.00) |
| Mg | 0.960 | 0 (0.00) |
| Al | 0.610 | 70 (24.31) |
| Ti | 6.670 | 1 (0.35) |
| V | 0.090 | 1 (0.35) |
| Mn | 0.130 | 12 (4.17) |
| Fe | 8.350 | 12 (4.17) |
| Co | 0.003 | 0 (0.00) |
| Ni | 0.015 | 6 (2.08) |
| Cu | 0.51 | 69 (23.96) |
| Zn | 0.47 | 14 (4.86) |
| As | 0.110 | 0 (0.00) |
| Rb | 0.020 | 0 (0.00) |
| Sr | 0.100 | 0 (0.00) |
| Mo | 0.020 | 0 (0.00) |
| Te | 0.002 | 49 (17.01) |
| I | 0.590 | 20 (6.94) |

The units of metals are micrograms per liter (μg/L).

LOD: limit of detection, B: boron, Mg: magnesium, Al: aluminum, Ti: titanium, V: vanadium, Mn: manganese, Fe: iron, Co: cobalt, Ni: nickel, Cu: copper, Zn: zinc, As: arsenic, Rb: rubidium, Sr: strontium, Mo: molybdenum, Te: tellurium, I: iodine.

**Table S2.** Concentrations of elements among study participants (n=288)

| Elements | Group | P_25_ | P_50_ | P_75_ | p value |
| --- | --- | --- | --- | --- | --- |
| B | Case | 385.53 | 521.74 | 688.73 | 0.533 |
|  | Control | 324.46 | 563.24 | 955.21 |  |
| Mg | Case | 36103.54 | 54010.40 | 75714.72 | 0.446 |
|  | Control | 26132.80 | 51388.48 | 80183.34 |  |
| Al | Case | 1.18 | 2.81 | 7.73 | 0.534 |
|  | Control | 0.85 | 3.67 | 10.10 |  |
| Ti | Case | 876.03 | 1379.12 | 1875.35 | 0.001 |
|  | Control | 1009.57 | 1768.54 | 2988.29 |  |
| V | Case | 16.86 | 28.91 | 49.91 | 0.493 |
|  | Control | 15.66 | 26.32 | 42.85 |  |
| Mn | Case | 0.48 | 0.77 | 1.43 | < 0.001 |
|  | Control | 0.86 | 2.09 | 3.99 |  |
| Fe | Case | 205.42 | 367.53 | 704.74 | 0.562 |
|  | Control | 165.69 | 344.43 | 713.00 |  |
| Co | Case | 0.30 | 0.49 | 0.85 | 0.780 |
|  | Control | 0.26 | 0.52 | 0.98 |  |
| Ni | Case | 1.65 | 2.51 | 3.78 | 0.710 |
|  | Control | 1.25 | 2.37 | 4.58 |  |
| Cu | Case | 1.69 | 7.14 | 10.09 | 0.482 |
|  | Control | 1.06 | 7.20 | 15.21 |  |
| Zn | Case | 105.76 | 167.57 | 275.51 | 0.826 |
|  | Control | 80.88 | 172.31 | 366.90 |  |
| As | Case | 19.43 | 31.29 | 48.75 | 0.631 |
|  | Control | 19.27 | 30.89 | 45.81 |  |
| Rb | Case | 547.87 | 711.62 | 936.12 | < 0.001 |
|  | Control | 622.79 | 947.86 | 1578.33 |  |
| Sr | Case | 72.79 | 110.99 | 176.05 | 0.48 |
|  | Control | 60.75 | 105.14 | 196.05 |  |
| Mo | Case | 36.19 | 37.49 | 56.71 | 0.487 |
|  | Control | 23.27 | 38.32 | 74.84 |  |
| Te | Case | 0.02 | 0.04 | 0.06 | 0.035 |
|  | Control | 0.02 | 0.04 | 0.08 |  |
| I | Case | 79.30 | 159.04 | 246.47 | < 0.001 |
|  | Control | 159.12 | 279.02 | 504.66 |  |

The units of trace elements were micrograms per gram creatinine (μg/g).

B: boron, Mg: magnesium, Al: aluminum, Ti: titanium, V: vanadium, Mn: manganese, Fe: iron, Co: cobalt, Ni: nickel, Cu: copper, Zn: zinc, As: arsenic, Rb: rubidium, Sr: strontium, Mo: molybdenum, Te: tellurium, I: iodine.

**Fig. S1** The Spearman correlation heatmap of urinary elements’ levels (log) among the overall participants.


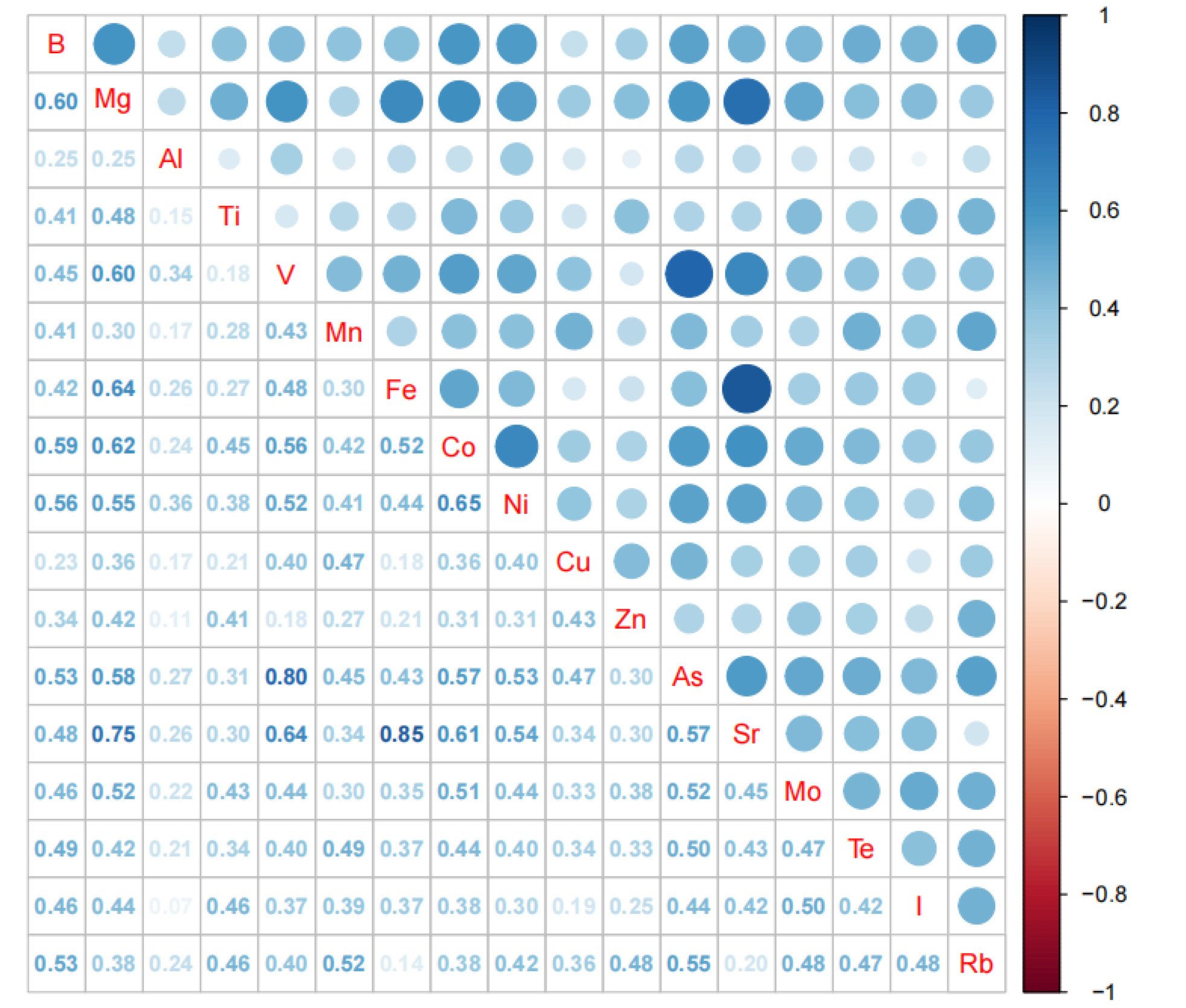


B: boron, Mg: magnesium, Al: aluminum, Ti: titanium, V: vanadium, Mn: manganese, Fe: iron, Co: cobalt, Ni: nickel, Cu: copper, Zn: zinc, As: arsenic, Rb: rubidium, Sr: strontium, Mo: molybdenum, Te: tellurium, I: iodine.

**Table S3.** Concentrations of elements among Depression and Manic (n=144).

| Elements | Group | P_25_ | P_50_ | P_75_ | p value |
| --- | --- | --- | --- | --- | --- |
| B | Depression | 389.28 | 518.36 | 687.27 | 0.747 |
|  | Manic | 383.84 | 529.09 | 703.34 |  |
| Mg | Depression | 36243.27 | 54274.78 | 76005.51 | 0.747 |
|  | Manic | 35892.74 | 52407.39 | 75753.59 |  |
| Al | Depression | 1.16 | 2.79 | 7.07 | 0.478 |
|  | Manic | 1.18 | 3.08 | 8.55 |  |
| Ti | Depression | 877.70 | 1429.23 | 1874.52 | 0.826 |
|  | Manic | 854.80 | 1328.83 | 1908.71 |  |
| V | Depression | 15.29 | 25.89 | 45.78 | 0.094 |
|  | Manic | 18.85 | 32.53 | 56.11 |  |
| Mn | Depression | 0.47 | 0.77 | 1.31 | 0.698 |
|  | Manic | 0.48 | 0.76 | 1.50 |  |
| Fe | Depression | 223.14 | 388.55 | 664.41 | 0.441 |
|  | Manic | 171.41 | 305.77 | 779.15 |  |
| Co | Depression | 0.28 | 0.52 | 0.85 | 0.979 |
|  | Manic | 0.30 | 0.47 | 0.85 |  |
| Ni | Depression | 1.49 | 2.47 | 3.85 | 0.650 |
|  | Manic | 1.74 | 2.53 | 3.57 |  |
| Cu | Depression | 0.89 | 6.79 | 9.82 | 0.169 |
|  | Manic | 5.12 | 7.72 | 10.44 |  |
| Zn | Depression | 105.62 | 170.24 | 270.54 | 0.881 |
|  | Manic | 105.47 | 159.61 | 279.85 |  |
| As | Depression | 16.65 | 27.28 | 44.64 | 0.048 |
|  | Manic | 22.11 | 33.94 | 50.51 |  |
| Rb | Depression | 534.25 | 693.99 | 888.84 | 0.158 |
|  | Manic | 592.41 | 756.13 | 1169.01 |  |
| Sr | Depression | 71.93 | 111.15 | 178.04 | 0.966 |
|  | Manic | 73.19 | 109.10 | 171.33 |  |
| Mo | Depression | 24.65 | 35.25 | 53.21 | 0.293 |
|  | Manic | 27.93 | 42.77 | 63.04 |  |
| Te | Depression | 0.02 | 0.03 | 0.05 | 0.394 |
|  | Manic | 0.01 | 0.03 | 0.07 |  |
| I | Depression | 77.87 | 152.67 | 249.94 | 0.629 |
|  | Manic | 84.67 | 165.82 | 246.66 |  |

B: boron, Mg: magnesium, Al: aluminum, Ti: titanium, V: vanadium, Mn: manganese, Fe: iron, Co: cobalt, Ni: nickel, Cu: copper, Zn: zinc, As: arsenic, Rb: rubidium, Sr: strontium, Mo: molybdenum, Te: tellurium, I: iodine.

The units of trace elements were micrograms per gram creatinine (μg/g).

**Table S4.** Odds ratios (ORs) and 95% confidence intervals (95% CIs) for the manic phase versus the depressive phase based on urine element in single-element model (n=144).

| Elements | Quartile 1 | Quartile 2 | Quartile 3 | Quartile 4 | p trend |
| --- | --- | --- | --- | --- | --- |
| B |  |  |  |  |  |
| Range | ≤5.96 | ~6.25 | ~6.53 | ≥6.54 |  |
| Depression/Manic | 22/16 | 23/11 | 22/14 | 22/14 |  |
| Model 1 | 1.00(ref) | 0.76 (0.28-2.05) | 0.92 (0.36-2.38) | 1.03 (0.39-2.73) | 0.916 |
| Model 2 | 1.00 (ref) | 0.56 (0.18-1.78) | 0.66 (0.22-2.02) | 1.03 (0.34-3.08) | 0.969 |
| Mg |  |  |  |  |  |
| Range | ≤10.50 | ~10.90 | ~11.24 | ≥11.25 |  |
| Depression/Manic | 22/14 | 22/14 | 23/14 | 22/13 |  |
| Model 1 | 1.00 (ref) | 1.07 (0.41-2.78) | 0.93 (0.36-2.43) | 0.98 (0.37-2.58) | 0.918 |
| Model 2 | 1.00 (ref) | 0.95 (0.30-2.95) | 0.74 (0.25-2.24) | 0.58 (0.19-1.83) | 0.338 |
| Al |  |  |  |  |  |
| Range | ≤-.15 | ~1.03 | ~1.96 | ≥1.97 |  |
| Depression/Manic | 22/13 | 23/13 | 22/9 | 22/20 |  |
| Model 1 | 1.00 (ref) | 1.04 (0.39-2.78) | 0.66 (0.23-1.91) | 1.73 (0.67-4.48) | 0.355 |
| Model 2 | 1.00 (ref) | 0.88 (0.27-2.86) | 0.39 (0.10-1.48) | 1.21 (0.364.12) | 0.859 |
| Ti |  |  |  |  |  |
| Range | ≤6.78 | ~7.26 | ~7.54 | ≥7.55 |  |
| Depression/Manic | 22/14 | 23/17 | 22/10 | 22/14 |  |
| Model 1 | 1.00 (ref) | 1.17 (0.46-2.98) | 0.71 (0.26-1.96) | 1.04 (0.40-2.73) | 0.865 |
| Model 2 | 1.00 (ref) | 0.84 (0.26-2.66) | 0.75 (0.22-2.49) | 1.11 (0.34-3.56) | 0.938 |
| V |  |  |  |  |  |
| Range | ≤2.73 | ~3.25 | ~3.82 | ≥3.83 |  |
| Depression/Manic | 22/8 | 23/14 | 22/14 | 22/19 |  |
| Model 1 | 1.00 (ref) | 1.76 (0.61-5.07) | 1.66 (0.57-4.83) | 2.36 (0.84-6.64) | 0.133 |
| Model 2 | 1.00 (ref) | 1.39 (0.42-4.59) | 1.27 (0.37-4.28) | 1.79 (0.54-5.95) | 0.389 |
| Mn |  |  |  |  |  |
| Range | ≤-0.74 | ~-0.26 | ~0.27 | ≥0.28 |  |
| Depression/Manic | 22/12 | 22/16 | 23/10 | 22/17 |  |
| Model 1 | 1.00 (ref) | 1.41 (0.53-3.71) | 0.94 (0.33-2.69) | 1.77 (0.65-4.81) | 0.361 |
| Model 2 | 1.00 (ref) | 1.42 (0.47-4.28) | 0.57 (0.16-1.99) | 1.91 (0.60-6.09) | 0.443 |
| Fe |  |  |  |  |  |
| Range | ≤5.41 | ~5.96 | ~6.50 | ≥6.51 |  |
| Depression/Manic | 22/18 | 22/13 | 23/6 | 22/18 |  |
| Model 1 | 1.00 (ref) | 0.76 (0.29-1.97) | 0.38 (0.12-1.17) | 1.25 (0.49-3.18) | 0.858 |
| Model 2 | 1.00 (ref) | 0.85 (0.28-2.56) | 0.41 (0.11-1.47) | 1.25 (0.44-3.56) | 0.884 |
| Co |  |  |  |  |  |
| Range | ≤-1.25 | ~-0.65 | ~-0.16 | ≥-0.15 |  |
| Depression/Manic | 22/10 | 23/23 | 22/8 | 22/14 |  |
| Model 1 | 1.00 (ref) | 2.17 (0.82-5.72) | 0.96 (0.30-2.99) | 1.63 (0.56-4.78) | 0.702 |
| Model 2 | 1.00 (ref) | 2.01 (0.67-6.03) | 0.74 (0.19-2.80) | 1.34 (0.39-4.59) | 0.974 |
| Ni |  |  |  |  |  |
| Range | ≤0.40 | ~0.91 | ~1.35 | ≥1.36 |  |
| Depression/Manic | 22/8 | 22/19 | 23/15 | 22/13 |  |
| Model 1 | 1.00 (ref) | 2.24 (0.79-6.32) | 1.86 (0.64-5.41) | 1.86 (0.62-5.53) | 0.358 |
| Model 2 | 1.00 (ref) | 2.91 (0.83-10.27) | 2.10 (0.60-7.33) | 1.66 (0.45-6.14) | 0.629 |
| Cu |  |  |  |  |  |
| Range | ≤-0.11 | ~1.92 | ~2.29 | ≥2.30 |  |
| Depression/Manic | 22/8 | 23/12 | 22/19 | 22/16 |  |
| Model 1 | 1.00 (ref) | 1.50 (0.51-4.41) | 2.19 (0.78-6.13) | 2.01 (0.71-5.69) | 0.136 |
| Model 2 | 1.00 (ref) | 1.29 (0.35-4.84) | 1.93 (0.53-7.00) | 2.38 (0.62-9.19) | 0.216 |
| Zn |  |  |  |  |  |
| Range | ≤4.66 | ~5.14 | ~5.60 | ≥5.61 |  |
| Depression/Manic | 22/14 | 22/15 | 23/10 | 22/16 |  |
| Model 1 | 1.00 (ref) | 0.98 (0.38-2.53) | 0.62 (0.22-1.72) | 0.95 (0.36-2.49) | 0.738 |
| Model 2 | 1.00 (ref) | 0.67 (0.22-2.06) | 0.50 (0.15-1.68) | 0.77 (0.24-2.47) | 0.587 |
| As |  |  |  |  |  |
| Range | ≤2.81 | ~3.31 | ~3.80 | ≥3.81 |  |
| Depression/Manic | 22/4 | 22/13 | 23/19 | 22/19 |  |
| Model 1 | 1.00 (ref) | 3.09 (0.86-11.06) | 4.29 (1.24-14.92) | 4.56 (1.31-15.89) | 0.021 |
| Model 2 | 1.00 (ref) | 3.73 (0.87-15.94) | 6.72 (1.62-27.95) | 5.31 (1.20-23.45) | 0.029 |
| Rb |  |  |  |  |  |
| Range | ≤6.28 | ~6.54 | ~6.79 | ~6.80 |  |
| Depression/Manic | 22/9 | 23/15 | 22/11 | 22/20 |  |
| Model 1 | 1.00 (ref) | 2.13 (0.72-6.29) | 1.10 (0.37-3.30) | 2.59 (0.91-7.39) | 0.136 |
| Model 2 | 1.00 (ref) | 1.56 (0.45-5.39) | 0.72 (0.20-2.56) | 2.15 (0.64-7.24) | 0.254 |
| Sr |  |  |  |  |  |
| Range | ≤4.28 | ~4.71 | ~5.18 | ~5.19 |  |
| Depression/Manic | 22/13 | 23/15 | 22/16 | 22/11 |  |
| Model 1 | 1.00 (ref) | 1.09 (0.42-2.82) | 1.36 (0.52-3.54) | 0.96 (0.35-2.67) | 0.958 |
| Model 2 | 1.00 (ref) | 1.17 (0.39-3.50) | 1.26 (0.42-3.75) | 0.81 (0.25-2.59) | 0.788 |
| Mo |  |  |  |  |  |
| Range | ≤3.20 | ~3.56 | ~3.97 | ≥3.98 |  |
| Depression/Manic | 22/11 | 22/11 | 23/15 | 22/18 |  |
| Model 1 | 1.00 (ref) | 1.04 (0.37-2.91) | 1.25 (0.47-3.35) | 1.58 (0.60-4.14) | 0.318 |
| Model 2 | 1.00 (ref) | 1.24 (0.35-4.39) | 1.46 (0.46-4.61) | 1.36 (0.43-4.26) | 0.581 |
| Te |  |  |  |  |  |
| Range | ≤-3.89 | ~-3.37 | ~-2.95 | ≥-2.94 |  |
| Depression/Manic | 22/15 | 22/11 | 23/10 | 22/19 |  |
| Model 1 | 1.00 (ref) | 0.84 (0.31-2.28) | 0.76 (0.27-2.09) | 1.61 (0.62-4.16) | 0.316 |
| Model 2 | 1.00 (ref) | 0.46 (0.14-1.53) | 0.54 (0.16-1.75) | 1.14 (0.38-3.46) | 0.665 |
| I |  |  |  |  |  |
| Range | ≤4.36 | ~5.03 | ~5.52 | ≥5.53 |  |
| Depression/Manic | 22/12 | 22/13 | 23/17 | 22/13 |  |
| Model 1 | 1.00 (ref) | 1.14 (0.42-3.08) | 1.34 (0.52-3.46) | 1.08 (0.40-2.91) | 0.703 |
| Model 2 | 1.00 (ref) | 2.16 (0.63-7.39) | 2.83 (0.86-9.36) | 1.49 (0.46-4.86) | 0.216 |

Model 1: Only adjusted by gender and age (continuous variable).

Model 2: Additionally adjusted for covariates: BMI, residence, monthly household income, physical activity, sleep, smoking, alcohol consumption, family history of mental diseases.

B: boron, Mg: magnesium, Al: aluminum, Ti: titanium, V: vanadium, Mn: manganese, Fe: iron, Co: cobalt, Ni: nickel, Cu: copper, Zn: zinc, As: arsenic, Rb: rubidium, Sr: strontium, Mo: molybdenum, Te: tellurium, I: iodine.

**Table S5.** Multi-element model for adolescent bipolar disorder depression phase associated with multiple elements and characteristics of participants (n=234).

| Elements | Quartile 1 | Quartile 2 | Quartile 3 | Quartile 4 | p-trend |
| --- | --- | --- | --- | --- | --- |
| Mg | 1.00 (ref) | 2.75 (0.83-9.16) | 4.36 (1.17-16.25) | 8.77 (1.68-45.90) | 0.006 |
| Mn | 1.00 (ref) | 0.57 (0.25-1.31) | 0.13 (0.04-0.41) | 0.08 (0.02-0.30) | < 0.001 |
| Fe | 1.00 (ref) | 0.99 (0.28-3.52) | 1.48 (0.32-6.88) | 1.73 (0.25-11.93) | 0.349 |
| Sr | 1.00 (ref) | 1.07 (0.32-3.51) | 1.24 (0.27-5.57) | 1.91 (0.24-15.28) | 0.799 |
| I | 1.00 (ref) | 0.40 (0.17-0.97) | 0.12 (0.04-0.39) | 0.05 (0.01-0.24) | < 0.001 |

Adjusted for gender, age, BMI, residence, monthly household income, physical activity, sleep, smoking, alcohol consumption, family history of mental diseases and five element. Mg: magnesium, Mn: manganese, Fe: iron, Sr: strontium, I: iodine.


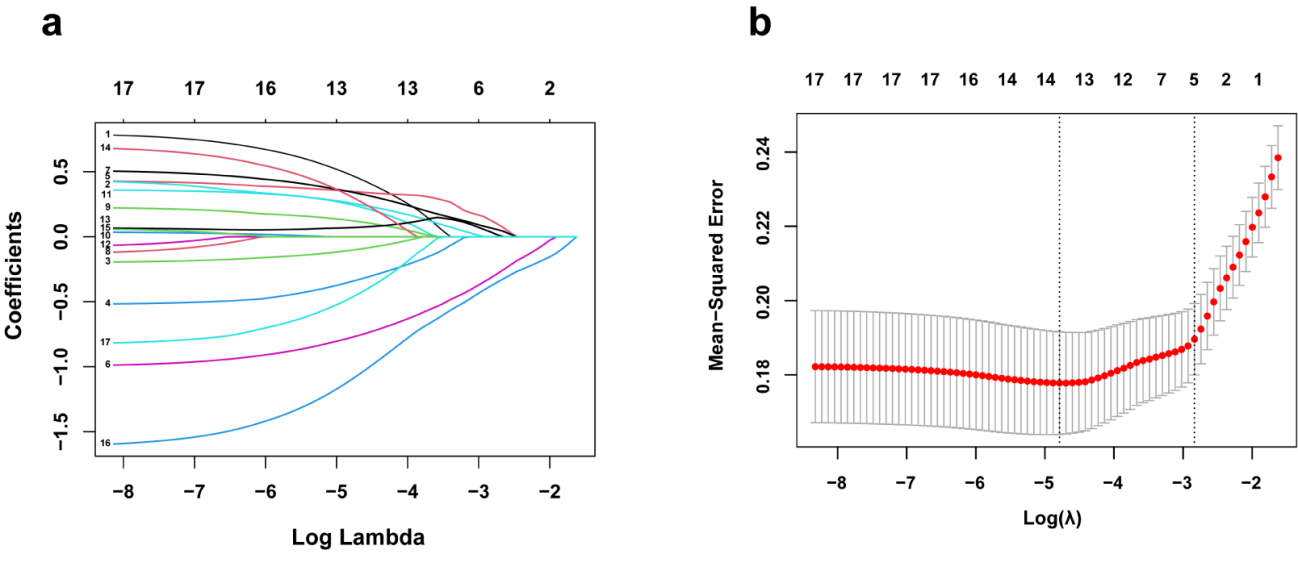


**Fig. S2** The elements of depressive phase selected into the multi-element model by LASSO regression. (a) The change in trajectory of each variable coefficient (λ). (b) Cross-validation plot for the penalty term.


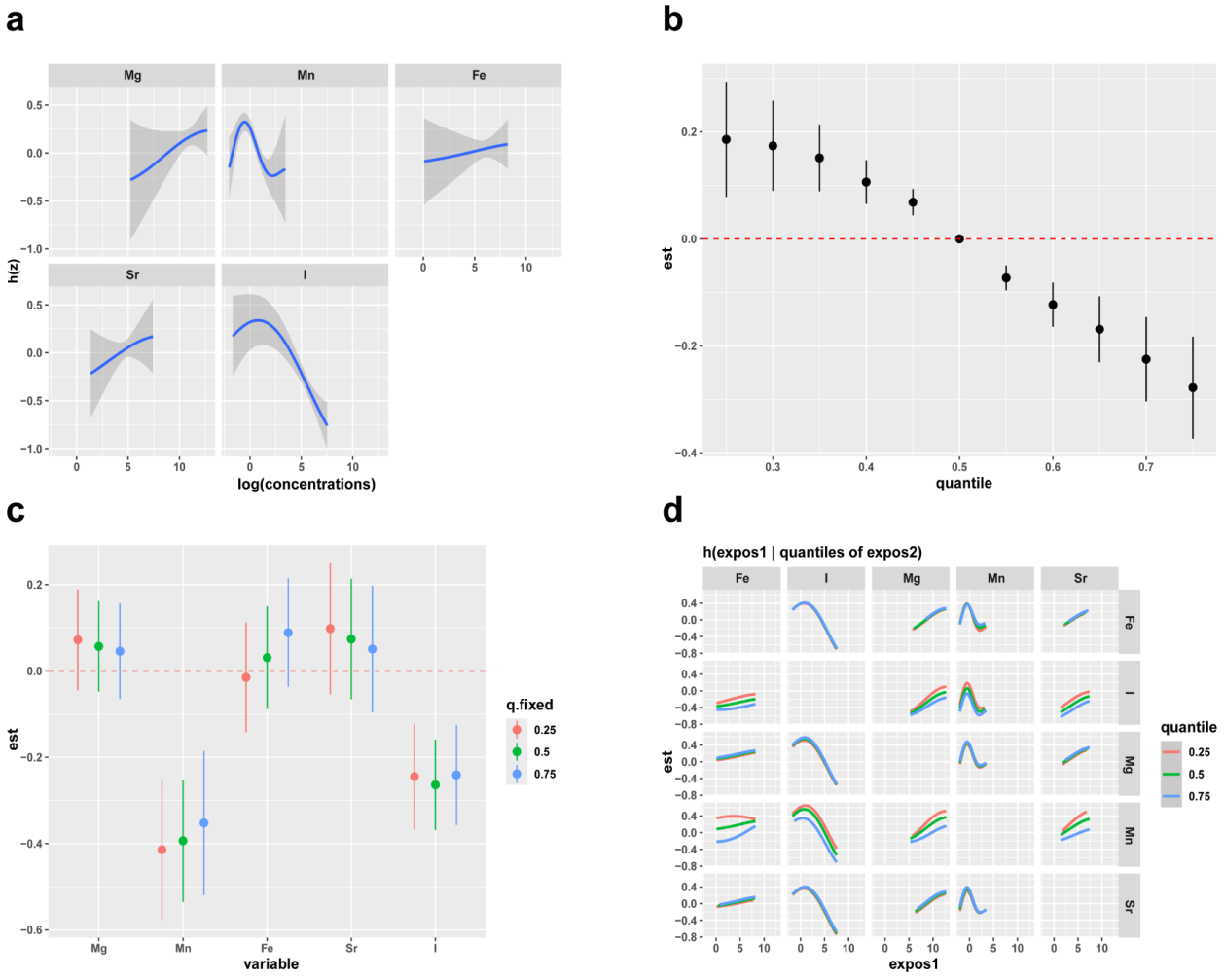


**Fig. S3** The BKMR models of each element and mixed exposure effects on depression phase. (a) Univariate exposure-response functions of each element (95%CI) with other elements fixed at their medians (P50). (b) The overall effects of mixed exposure, with metals fixed at different percentiles compared to their concentrations when they are at their medians (P50). (c) The effects of single-exposure when an individual element is at its 75th percentile as compared to when that exposure was at its 25th percentile, where all of other exposures are fixed to P75. (d) The bivariate cross-section effects of the exposure-response function of a single element where the second element is fixed at P25, P50 and P75.

**Table S6.** Multi-element model for adolescent bipolar disorder manic phase associated with multiple elements and characteristics of participants (n=200).

| Elements | Quartile 1 | Quartile 2 | Quartile 3 | Quartile 4 | p-trend |
| --- | --- | --- | --- | --- | --- |
| V | 1.00 (ref) | 0.91 (0.20-4.09) | 2.93 (0.59-14.56) | 31.50 (4.04-245.39) | 0.100 |
| Mn | 1.00 (ref) | 0.25 (0.07-0.89) | 0.01 (0.00-0.10) | 0.00 (0.00-0.02) | < 0.001 |
| Cu | 1.00 (ref) | 1.61 (0.37-6.92) | 20.38 (3.46-120.10) | 25.07 (1.35-464.95) | 0.708 |
| Sr | 1.00 (ref) | 0.82 (0.18-3.61) | 0.55 (0.08-3.50) | 2.23 (0.20-26.17) | 0.896 |
| I | 1.00 (ref) | 0.45 (0.11-1.78) | 0.10 (0.02-0.56) | 0.02 (0.01-0.22) | < 0.001 |

Adjusted for gender, age, BMI, residence, monthly household income, physical activity, sleep, smoking, alcohol consumption, family history of mental diseases and five element. V: vanadium, Mn: manganese, Cu: copper, Sr: strontium, I: iodine.


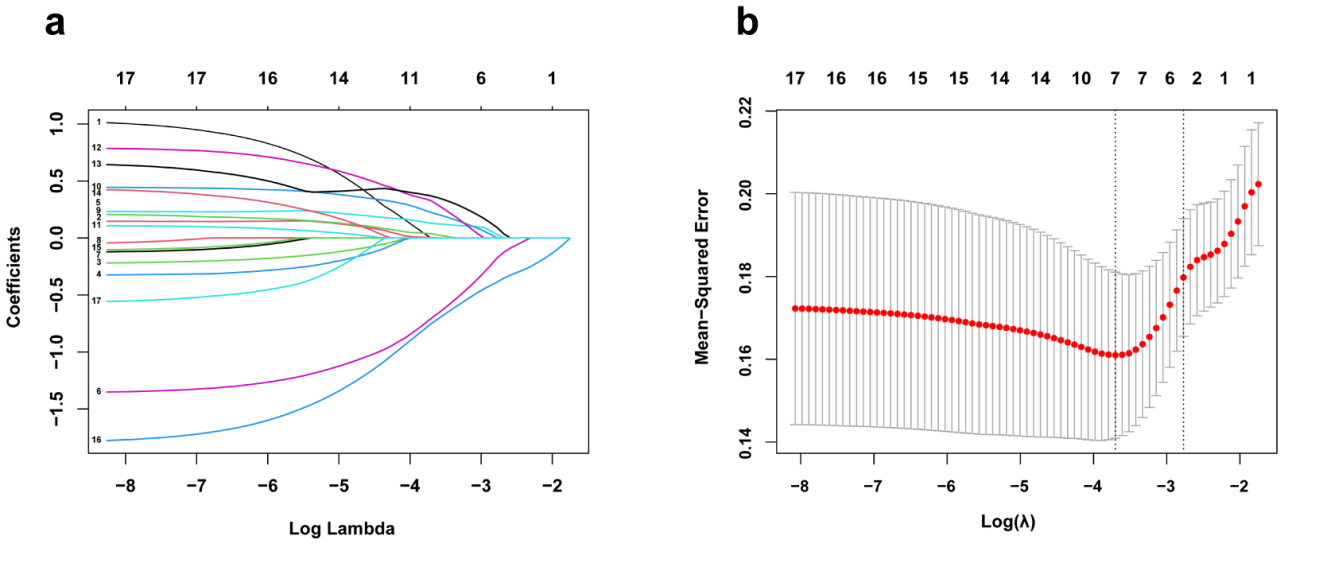


**Fig. S4.** The elements of manic phase selected into the multi-element model by LASSO regression. (a) The change in trajectory of each variable coefficient (λ). (b) Cross-validation plot for the penalty term.


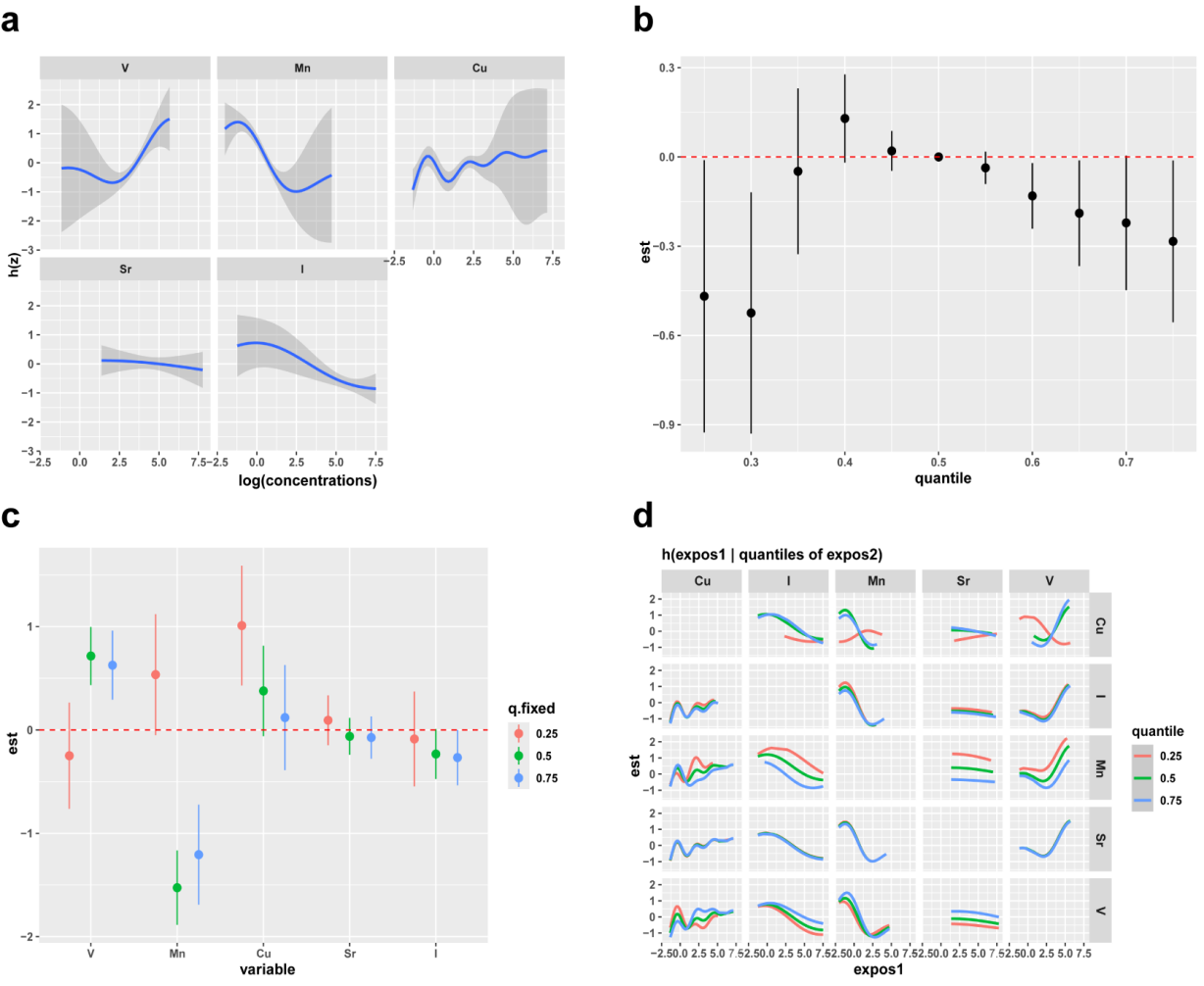


**Fig. S5.** The BKMR models of each element and mixed exposure effects on manic phase. (a) Univariate exposure-response functions of each element (95%CI) with other elements fixed at their medians (P50). (b) The overall effects of mixed exposure, with metals fixed at different percentiles compared to their concentrations when they are at their medians (P50). (c) The effects of single-exposure when an individual element is at its 75th percentile as compared to when that exposure was at its 25th percentile, where all of other exposures are fixed to P75. (d) The bivariate cross-section effects of the exposure-response function of a single element where the second element is fixed at P25, P50 and P75.

**Table S7.** Clinical trials registration of bipolar disorder and trace elements

| Trial ID | Trial name | Status | Study start | Study completion | Enrollment | Study objective | Research institution |
| --- | --- | --- | --- | --- | --- | --- | --- |
| NCT05837104 | Efficacy and Safety of Magnesium Vitamin B6 in First Episode Bipolar Disorder | Recruiting | 2023-12 | 2025-12 | 40 | Evaluating magnesium-B6 with standard treatment for depression, stress, and anxiety in first-episode bipolar I patients. | Mclean Hospital, America |
| NCT03541031 | Micronutrients as Adjunctive Treatment for Bipolar Disorder | Recruiting | 2018-05 | 2019-06 | 120 | Evaluating micronutrients for bipolar treatment and reduced side effects. | Eastern Maine Medical Center, America |
| NCT02986490 | Magnesium Variations and Cardiometabolic Risk in Patients With Antipsychotic Drugs | Recruiting | 2014-09 | 2017-12 | 100 | Mg levels and cardiometabolic risk during antipsychotic drug use in patients with BD. | University Hospital, Montpellier, France |
| NCT02081287 | Inositol Hexaphosphate: A Novel Treatment Strategy for Bipolar Disorder? | Completed | 2014-05 | 2018-07 | 30 | Efficacy of IP6 calcium-magnesium salt in BD treatment. | San Diego Veterans Healthcare System, America |
| NCT00109577 | Clinical Trial of a Nutritional Supplement in Adults With Bipolar Disorder | Terminated | 2005-04 | 2009-03 | 40 | Evaluating a 36-ingredient micronutrient supplement for BD. | University of Calgary, Canada |

**Table S8.** Clinical studies on bipolar disorder and trace elements

| First author (year) | Study type | Study population (age [years]) | Sample size  (BD/Control) | Trace element(s) | Detection method | Main finding |
| --- | --- | --- | --- | --- | --- | --- |
| Jonsson (2022) | Clinical study | 45-50 | 121/30 | Zn | Colorimetric method | BD patients had higher serum Zn levels. |
| Santa Cruz (2020) | Clinical study | 30-36 | 15/11 | Se, Zn, Fe, K, Ca, Mg, P, Al, Cu, Mn, Ni | ICP-MS | Se and Zn were significantly lower in BD patients. |
| Steardo (2020) | Clinical study | 18-65 | 199/0 | Ca | Standard Lab | Abnormal Ca homeostasis in BD. |
| Styczen (2018) | Clinical study | 18-65 | 110/114 | Zn, Cu | FAAS, ETAAS | No significant Zn and Cu differences between BD and MDD patients. |
| Tarleton (2017) | Clinical study | Mean: 52 | 126/0 | Mg | Not specified | Mg was effective for mild-to-moderate BD depression. |
| Chowdhury (2017) | Clinical study | Mean: 25 | 55/55 | Zn, Fe, Se, Na, K, Ca | FAAS, GFAAS | BD patients had lower Zn, Fe, Se, Na, K, and Ca levels. |
| Millett (2017) | Clinical study | 19-58 | 27/31 | Zn, neopterin | FAAS | Mania severity was associated with neopterin in men, depression severity was positively associated with Zn in women. |
| Siwek (2017) | Clinical study | 18-65 | 133/50 | Cu | FAAS | Higher Cu in early-stage BD, lower in later-stage BD. |
| Siwek (2016) | Clinical study | 18-65 | 129/50 | Zn | FAAS | Zn levels were lower in BD I depressive episodes. |
| Siwek (2015) | Clinical study | 18-65 | 129/50 | Mg | FAAS | Serum Mg was higher in depressive and manic states. |
| Dubovsky (2014) | Clinical study | Mean: 40 | 15/17 | Ca²⁺ | Not specified | Higher platelet Ca²⁺ levels in BD. |
| Gonzalez-Estecha (2011) | Clinical study | Mean: 49 | 25/29 | Cu, Zn, Pb, Cd, Tl | FAAS, ICP-MS | BD patients had higher Pb, Cd than the control. Manic patients had higher Zn than controls. |
| Mustak (2008) | Clinical study | 21-42 | 115/25 | Na, K, S, Ca, Mg, P, Cu, Fe, Zn, Mn, Al | ICP-AES | In bipolar I (mania), Na, K, P, Cu, Al, and Mn increased significantly. In bipolar II hypomania, Na, S, Al, and Mn increased significantly). In bipolar II depression, Na, K, Cu, and Al increased. |
| Nechifor (2023) | Review | - | - | Mg, Zn | - | Low level of Mg and Zn play a role in pathogenesis of BD. |
| Sylvia (2013) | Review | - | - | Choline, Mg, Folate, Tryptophan | - | Choline, Mg, folate, and tryptophan may reduce manic symptoms. |

FAAS: Flame atomic absorption spectrometry, ETAAS: Electrothermal atomic absorption spectrometry, ICP-AES: Inductively coupled plasma-atomic emission spectrometry, GFAAS: Graphite furnace atomic absorption spectrometry, ICP-MS: Inductively coupled plasma mass spectrometry.

**Table S9.** Basic studies on bipolar disorder and trace elements

| First author (year) | Animals | Special treatments | Other research technologies | Results or conclusions |
| --- | --- | --- | --- | --- |
| Huzayyin (2024) | Male C57BL/6 mice | Zn, VitD3 feeding | Western Blot, ELISA, Colorimetric assay, Spectrophotometric | Zn, VitD3 supplementation may improve the treatment of BD. |
| Caffino (2024) | Male Wistar rats | Li injection | Western Blot, Neuronal label, Morphological classification | Li can reduce glutamate hyperexcitability in the manic phase of BD. |
| Zhang (2023) | Male C57BL/6J mice | Cu in drinking water | Western Blot, ELISA, Immunochemistry, TUNEL | Cu causes neuronal damage and cognitive deficits in manic mice via cuproptosis and synaptic dysfunction. |
| Valvassori (2019) | Male Wistar rats | Li in drinking water | Western Blot, Enzyme assay | Li reverses manic-like behavior in BD and reduces oxidative stress. |
| Zhu (2012) | Male SD rats | Zn injection | Western Blot, Immunocytochemistry | Zn disrupts NMDA receptor function in hippocampal neurons, reducing excitability in BD. |

ELISA: Enzyme linked immunosorbent assay, TUNEL: Terminal-deoxynucleoitidyl transferase mediated Nick end labeling, SD: Sprague-Dawley, NMDA: N-methyl-D-aspartic acid receptor.

**Reference**

Jonsson BH, Orhan F, Bruno S, Oliveira AO, Sparding T, Landen M, et al. Serum concentration of zinc is elevated in clinically stable bipolar disorder patients. Brain Behav. 2022;12(1):e2472. Epub 2021/12/31. doi: 10.1002/brb3.2472. PubMed PMID: 34967503; PubMed Central PMCID: PMCPMC8785612.

Santa Cruz EC, Madrid KC, Arruda MAZ, Sussulini A. Association between trace elements in serum from bipolar disorder and schizophrenia patients considering treatment effects. J Trace Elem Med Biol. 2020;59:126467. Epub 2020/01/20. doi: 10.1016/j.jtemb.2020.126467. PubMed PMID: 31954929.

Steardo L, Jr., Luciano M, Sampogna G, Carbone EA, Caivano V, Di Cerbo A, et al. Clinical Severity and Calcium Metabolism in Patients with Bipolar Disorder. Brain Sci. 2020;10(7). Epub 2020/07/08. doi: 10.3390/brainsci10070417. PubMed PMID: 32630307; PubMed Central PMCID: PMCPMC7408522.

Styczen K, Sowa-Kucma M, Dudek D, Siwek M, Reczynski W, Szewczyk B, et al. Zinc and copper concentration do not differentiate bipolar disorder from major depressive disorder. Psychiatr Pol. 2018;52(3):449-57. Epub 2018/09/16. doi: 10.12740/PP/OnlineFirst/80069. PubMed PMID: 30218561.

Tarleton EK, Littenberg B, MacLean CD, Kennedy AG, Daley C. Role of magnesium supplementation in the treatment of depression: A randomized clinical trial. PLoS One. 2017;12(6):e0180067. Epub 2017/06/28. doi: 10.1371/journal.pone.0180067. PubMed PMID: 28654669; PubMed Central PMCID: PMCPMC5487054.

Chowdhury MI, Hasan M, Islam MS, Sarwar MS, Amin MN, Uddin SMN, et al. Elevated serum MDA and depleted non-enzymatic antioxidants, macro-minerals and trace elements are associated with bipolar disorder. J Trace Elem Med Biol. 2017;39:162-8. Epub 2016/12/03. doi: 10.1016/j.jtemb.2016.09.012. PubMed PMID: 27908410.

Millett CE, Mukherjee D, Reider A, Can A, Groer M, Fuchs D, et al. Peripheral zinc and neopterin concentrations are associated with mood severity in bipolar disorder in a gender-specific manner. Psychiatry Res. 2017;255:52-8. Epub 2017/05/22. doi: 10.1016/j.psychres.2017.05.022. PubMed PMID: 28528241; PubMed Central PMCID: PMCPMC5545151.

Siwek M, Styczen K, Sowa-Kucma M, Dudek D, Reczynski W, Szewczyk B, et al. The serum concentration of copper in bipolar disorder. Psychiatr Pol. 2017;51(3):469-81. Epub 2017/09/04. doi: 10.12740/PP/OnlineFirst/65250. PubMed PMID: 28866717.

Siwek M, Sowa-Kucma M, Styczen K, Szewczyk B, Reczynski W, Misztak P, et al. Decreased serum zinc concentration during depressive episode in patients with bipolar disorder. J Affect Disord. 2016;190:272-7. Epub 2015/11/06. doi: 10.1016/j.jad.2015.10.026. PubMed PMID: 26540081.

Siwek M, Styczen K, Sowa-Kucma M, Dudek D, Reczynski W, Szewczyk B, et al. The serum concentration of magnesium as a potential state marker in patients with diagnosis of bipolar disorder. Psychiatr Pol. 2015;49(6):1277-87. Epub 2016/02/26. doi: 10.12740/PP/OnlineFirst/42047. PubMed PMID: 26909402.

Dubovsky SL, Daurignac E, Leonard KE. Increased platelet intracellular calcium ion concentration is specific to bipolar disorder. J Affect Disord. 2014;164:38-42. Epub 2014/05/27. doi: 10.1016/j.jad.2014.04.025. PubMed PMID: 24856551.

Gonzalez-Estecha M, Trasobares EM, Tajima K, Cano S, Fernandez C, Lopez JL, et al. Trace elements in bipolar disorder. J Trace Elem Med Biol. 2011;25 Suppl 1:S78-83. Epub 2011/01/19. doi: 10.1016/j.jtemb.2010.10.015. PubMed PMID: 21242074.

Mustak MS, Rao TS, Shanmugavelu P, Sundar NM, Menon RB, Rao RV, et al. Assessment of serum macro and trace element homeostasis and the complexity of inter-element relations in bipolar mood disorders. Clin Chim Acta. 2008;394(1-2):47-53. Epub 2008/05/07. doi: 10.1016/j.cca.2008.04.003. PubMed PMID: 18457668.

Nechifor M. Magnesium and Zinc in Bipolar Disorders. Biomedical & Pharmacology Journal. 2023;Vol. 16(1), p. 1-14.

Sylvia LG, Peters AT, Deckersbach T, Nierenberg AA. Nutrient-based therapies for bipolar disorder: a systematic review. Psychother Psychosom. 2013;82(1):10-9. Epub 2012/11/14. doi: 10.1159/000341309. PubMed PMID: 23147067.

Huzayyin AAS, Ibrahim MK, Hassanein NMA, Ahmed HMS. Vitamin D3 and zinc supplements augment the antimanic efficacy of lithium and olanzapine treatments in an animal model of mania. Nutr Neurosci. 2024;27(12):1391-404. Epub 2024/04/18. doi: 10.1080/1028415X.2024.2338344. PubMed PMID: 38635860.

Caffino L, Targa G, Mallien AS, Mottarlini F, Rizzi B, Homberg JR, et al. Chronic Lithium Treatment Alters NMDA and AMPA Receptor Synaptic Availability and Dendritic Spine Organization in the Rat Hippocampus. Curr Neuropharmacol. 2024;22(12):2045-58. Epub 2023/09/15. doi: 10.2174/1570159X21666230913144420. PubMed PMID: 37711124; PubMed Central PMCID: PMCPMC11333793.

Zhang Y, Zhou Q, Lu L, Su Y, Shi W, Zhang H, et al. Copper Induces Cognitive Impairment in Mice via Modulation of Cuproptosis and CREB Signaling. Nutrients. 2023;15(4). Epub 2023/02/26. doi: 10.3390/nu15040972. PubMed PMID: 36839332; PubMed Central PMCID: PMCPMC9958748.

Valvassori SS, Tonin PT, Dal-Pont GC, Varela RB, Cararo JH, Garcia AF, et al. Coadministration of lithium and celecoxib reverses manic-like behavior and decreases oxidative stress in a dopaminergic model of mania induced in rats. Transl Psychiatry. 2019;9(1):297. Epub 2019/11/15. doi: 10.1038/s41398-019-0637-9. PubMed PMID: 31723123; PubMed Central PMCID: PMCPMC6853972

Zhu J, Shao CY, Yang W, Zhang XM, Wu ZY, Zhou L, et al. Chronic zinc exposure decreases the surface expression of NR2A-containing NMDA receptors in cultured hippocampal neurons. PLoS One. 2012;7(9):e46012. Epub 2012/10/11. doi: 10.1371/journal.pone.0046012. PubMed PMID: 23049922; PubMed Central PMCID: PMCPMC3457937.
