# Supplementary material for: Associations of multiple trace elements with bipolar disorder in adolescents: A case-control study
Source: PLoS One. 2025 May 6;20(5):e0322958. doi: 10.1371/journal.pone.0322958 (PMC12054906; doi:10.1371/journal.pone.0322958)
Supplement: Attachment — Submitted filename: Response to Reviewers.docx [file pone.0322958.s003.docx]

**REVIEWER' GENERAL POINTS:**

Reviewer #1: Your manuscript is highly interesting, as it provides valuable insights into elements that contribute to the diagnosis, treatment, and overall management of psychiatric conditions. Expanding our knowledge in this area is essential.

**Authors’ response**:

Thank you very much for your positive and helpful comments on our efforts. We have carefully revised the paper according to your suggestions point by point.

Major points 1: Did the BD participants receive pharmacological treatment? Could this treatment have influenced the urinary elements?

**Authors’ response**:

Thank you for your valuable comments. In our study, 139 patients with bipolar disorder were receiving pharmacological treatment, and we added the relevant content in Table 1. While pharmacological treatment can influence trace element levels - either increasing or decreasing specific elements - it is not considered the primary factor the observed changes in urinary trace element concentrations. We have expanded the discussion to reflect this, with the following addition: "*While pharmacological treatment can influence trace element levels - either increasing or decreasing specific elements - it is not considered the primary factor the observed changes in urinary trace element concentrations. For example, antipsychotic medications may partially explain fluctuations in trace element levels (Cu, Fe, etc.), either increasing or decreasing them (Wolf et al. 2006, Kabzińska-Milewska et al. 2021). However, other study found that the levels of elements (Mn, Mg, Zn, etc) in bipolar patients after treatment did not differ significantly from those in healthy individuals(Sampath et al. 2022, Rog et al. 2024).*" (see line 412 in the revised manuscript).

We also acknowledge the following limitations in our study: "*Firstly, our study did not specifically assess the potential influence of pharmacological treatment or lifestyle factors (such as diet, exercise, and smoking) on urinary trace element concentrations. These factors may affect trace element levels. Future research should aim to investigate these influences more directly, providing a clearer understanding of their role in trace element alterations in BD.*" (see line 428 in the revised manuscript).

**Table 1.** Baseline characteristics of cases and controls.

| Characteristics | Case (n = 144) | Control (n = 144) | p value |
| --- | --- | --- | --- |
| Girls, n (%) | 106 (73.60) | 106 (73.60) | 1.000 |
| Age, years, mean (SD) | 15.27 ± 0.14 | 15.10 ± 0.14 | 0.580 |
| BMI, kg/m^2^, n (%) | 144 | 144 | 0.213 |
| <18.5 | 41 (28.40) | 47 (32.60) |  |
| 18.5-23.9 | 76 (52.80) | 80 (55.60) |  |
| 24.0-27.9 | 21 (14.60) | 10 (6.90) |  |
| ≥28.0 | 6 (4.20) | 7 (4.90) |  |
| Resident, n (%) | 144 | 144 | 0.235 |
| urban | 137 (95.10) | 132 (91.70) |  |
| rural | 7 (4.90) | 12 (8.30) |  |
| Household monthly income, RMB, n (%) | 144 | 144 | 0.454 |
| ≤3000 | 23 (16.00) | 15 (10.40) |  |
| 3000-6000 | 71 (49.30) | 69 (47.90) |  |
| 6000-12000 | 35 (24.30) | 42 (29.20) |  |
| >12000 | 15 (10.40) | 18 (12.50) |  |
| Leisure time physical activity per  week, h, n (%) | 144 | 144 | 0.009 |
| <1 | 36 (25.00) | 25 (17.40) |  |
| 1-3 | 46 (31.90) | 70 (48.60) |  |
| 3-5 | 29 (20.20) | 33 (22.90) |  |
| 5-7 | 18 (12.50) | 7 (4.80) |  |
| ≥7 | 15 (10.40) | 9 (6.30) |  |
| Daily sleep time, h, n (%) | 144 | 144 | 0.802 |
| <8 | 96 (66.70) | 98 (68.10) |  |
| ≥8 | 48 (33.30) | 46 (31.90) |  |
| Smoking status, n (%) | 144 | 144 | 0.643 |
| No | 133 (92.40) | 135 (93.80) |  |
| Yes | 11 (7.60) | 9 (6.20) |  |
| Alcohol consumption, n (%) | 144 | 144 | 0.312 |
| No | 133 (92.40) | 128 (88.90) |  |
| Yes | 11 (7.60) | 16 (11.10) |  |
| Family history of mental diseases, n (%) | 144 | 144 | 0.090 |
| No | 137 (95.10) | 142 (98.60) |  |
| Yes | 7 (4.90) | 2 (1.40) |  |
| Suicide, n (%) | 144 | 144 | <0.001 |
| No suicidal intent | 22 (15.30) | 139 (97.20) |  |
| Suicidal intent | 39 (27.10) | 3 (2.10) |  |
| Suicidal attempt | 23 (16.00) | 0 (0.00) |  |
| Suicide attempt survivor | 60 (41.70) | 2 (1.40) |  |
| NSSI, n (%) | 144 | 144 | <0.001 |
| No | 47 (32.60) | 141 (97.90) |  |
| Yes | 97 (67.40) | 3 (2.10) |  |
| Current mood, n (%) | *144* |  |  |
| Manic | 55 (38.20) |  |  |
| Depression | 89 (61.80) |  |  |
| *Treatment, n (%)* | *144* |  |  |
| *No* | *5 (3.4)* |  |  |
| *Yes* | *139 (96.6)* |  |  |
| YMRS, mean (SD) | 10.87 ± 0.38 | 1.90 ± 0.14 | <0.001 |
| HAMD-17, mean (SD) | 17.17 ± 0.49 | 1.75 ± 0.17 | <0.001 |
| HAMA-14, mean (SD) | 11.23 ± 0.58 | 1.58 ± 0.14 | <0.001 |
| Urine creatinine, mean (SD) | 1.75 ± 0.08 | 1.59 ± 0.06 | 0.298 |

**Reference**

Wolf, T. L., J. Kotun and J. H. Meador-Woodruff (2006). "Plasma copper, iron, ceruloplasmin and ferroxidase activity in schizophrenia." Schizophr Res 86(1-3): 167-171.

Kabzińska-Milewska, K., R. W. Wójciak and D. J. J. o. E. Czajeczny (2021). "Effect of lithium treatment on the content of lithium, copper, calcium, magnesium, zinc and iron in the hair of patients with bipolar disorder."

Rog, J., L. Lobejko, M. Hordejuk, W. Marciniak, R. Derkacz, A. Kiljanczyk, M. Matuszczak, J. Lubinski, M. Nesterowicz, M. Zendzian-Piotrowska, A. Zalewska, M. Maciejczyk and H. Karakula-Juchnowicz (2024). "Pro/antioxidant status and selenium, zinc and arsenic concentration in patients with bipolar disorder treated with lithium and valproic acid." Front Mol Neurosci 17: 1441575.

Sampath, V. P., S. V. Singh, I. Pelov, O. Tirosh, Y. Erel and D. Lichtstein (2022). "Chemical Element Profiling in the Sera and Brain of Bipolar Disorders Patients and Healthy Controls." Int J Mol Sci 23(22).

**Major points 2:** Into the results, rewrite the sentences such as: "Significant differences were observed between the cases and controls in terms of physical activity, suicidal behavior, NSSI, YMRS, HAMD-17, and HAMA-14". It is not clear what are the differences between the two groups. Despite there is the table 1, it is preferable to write the results also in narrative form.

**Authors’ response**:

Thank you for your suggestion. We have revised the sentence to provide clearer. The revised version reads as follows: *“Significant differences were observed between the cases and control groups in physical activity (p = 0.009) and suicidal behavior (p < 0.001). In the case group, 67.4% of patients exhibited non-suicidal self-injury (NSSI) behavior, with the majority experiencing current mood depression (61.8%). Additionally, 96.6% of these patients were receiving treatment. Scores on the YMRS (p < 0.001), HAMD-17 (p < 0.001), and HAMA-14 (p < 0.001) were significantly higher in the case group compared to the control group.*" (see line 213 in the revised manuscript).

**Reviewer #2:** I have read the manuscript carefully, and my comments are as below:

**Authors’ response**:

Thanks very much for your careful reviewing of this manuscript and the constructive suggestion. We have carefully revised the paper according to your suggestions.

**Major points 1 and 2:** The title of the article "Associations of Multiple Trace Elements with the Risk of Bipolar Disorder in Adolescents: A Case-Control Study" is misleading, and it should be revised. How do you define "the risk of Bipolar Disorder"? This variable is not clearly defined in the manuscript.

**Authors’ response**:

Thank you for your suggestion. We agree with you, and we have revised the title to: "*Associations of Multiple Trace Elements with Bipolar Disorder in Adolescents: A Case-Control Study*." Meanwhile, we have thoroughly revised the manuscript to address any ambiguities, ensuring that all terms and variables are clearly defined.

**Major points 3 and 4:** In this work, 144 clinically defined BD subjects were selected along with the matching number of control normal subjects. The urine was analyzed for trace elements, and the statistical analysis was carried out. In plain words, there is a correlation between urinary elemental concentration among the BD subjects compared to normal subjects. Authors should comment on the possible reasons for elevated trace elements. Is it due to medication given to the BD subjects or due to food/water intake? The authors need to find out why there are differences in elemental concentrations.

**Authors’ response**:

Thank you for your insightful comments. According to your suggestion, we have expanded the discussion to address the potential causes of elevated trace elements in BD patients. In summary, the changes in urinary trace element levels in BD are likely influenced by a combination of neuroinflammation, neurotransmitter disturbances, oxidative stress, medication effects, altered gut microbiota, and dietary or environmental factors.

We have incorporated the following addition to reflect these factors more comprehensively: *"Individuals with BD often experience chronic neuroinflammation and disruptions in neurotransmitters systems such as dopamine and serotonin. The synthesis and metabolism of these neurotransmitters rely on multiple trace elements (Anderson et al. 2012).* *For example, trace elements like Zn and Mn are essential for the enzymatic reactions involved in dopamine and serotonin synthesis (Gao et al. 2024). Episodes of mania or depression can trigger oxidative stress and metabolic abnormalities, which may impair the absorption and utilization of trace elements (Shazia et al. 2012). Additionally, the gut microbiome in BD patients differs from that of healthy individuals, showing reduced microbial diversity and altered microbial abundance. This disruption can affect the absorption and utilization of trace elements, contributing to changes in their levels (Coello et al. 2019, Ortega et al. 2023). In conclusion, the causes of elevated trace elements in BD patients may be multifactorial, including altered neurotransmitter function, oxidative stress, and changes in gut microbiota composition."* (see line 401 in the revised manuscript).

Additionally, we discussed the influence of pharmacological treatments:“ *While pharmacological treatment can influence trace element levels - either increasing or decreasing specific elements - it is not considered the primary factor the observed changes in urinary trace element concentrations. For example, antipsychotic medications may partially explain fluctuations in trace element levels (Cu, Fe, etc.), either increasing or decreasing them (Wolf et al. 2006, Kabzińska-Milewska et al. 2021). However, other study found that the levels of elements (Mn, Mg, Zn, etc) in bipolar patients after treatment did not differ significantly from those in healthy individuals (Sampath et al. 2022, Rog et al. 2024). Moreover, while low dietary intake of Mn and Zn has been associated with symptoms of depression and anxiety, Mg intake did not show a significant relationship (Nakamura et al. 2019). A nationwide population-based study found no statistical significance between the lithium levels in drinking water and BD prevalence (Kessing et al. 2017).* *Therefore pharmacological treatment and dietary factors may influence trace element concentrations, these factors are not considered the primary causes of the observed changes in urinary trace element levels.*" (see line 412 in the revised manuscript).

Finally, We also acknowledge the following limitations in our study: "*Firstly, our study did not specifically assess the potential influence of pharmacological treatment or lifestyle factors (such as diet, exercise, and smoking) on urinary trace element concentrations. These factors may affect trace element levels. Future research should aim to investigate these influences more directly, providing a clearer understanding of their role in trace element alterations in BD.*" (see line 428 in the revised manuscript).

**Reference**

Anderson, I. M., P. M. Haddad and J. Scott (2012). "Bipolar disorder." BMJ 345: e8508.

Coello, K., T. H. Hansen, N. Sorensen, K. Munkholm, L. V. Kessing, O. Pedersen and M. Vinberg (2019). "Gut microbiota composition in patients with newly diagnosed bipolar disorder and their unaffected first-degree relatives." Brain Behav Immun 75: 112-118.

Gao, B., C. Li, Y. Qu, M. Cai, Q. Zhou, Y. Zhang, H. Lu, Y. Tang, H. Li and H. Shen (2024). "Progress and trends of research on mineral elements for depression." Heliyon 10(15): e35469.

Kabzińska-Milewska, K., R. W. Wójciak and D. J. J. o. E. Czajeczny (2021). "Effect of lithium treatment on the content of lithium, copper, calcium, magnesium, zinc and iron in the hair of patients with bipolar disorder."

Kessing, L. V., T. A. Gerds, N. N. Knudsen, L. F. Jorgensen, S. M. Kristiansen, D. Voutchkova, V. Ernstsen, J. Schullehner, B. Hansen, P. K. Andersen and A. K. Ersboll (2017). "Lithium in drinking water and the incidence of bipolar disorder: A nation-wide population-based study." Bipolar Disord 19(7): 563-567.

Nakamura, M., A. Miura, T. Nagahata, Y. Shibata, E. Okada and T. Ojima (2019). "Low Zinc, Copper, and Manganese Intake is Associated with Depression and Anxiety Symptoms in the Japanese Working Population: Findings from the Eating Habit and Well-Being Study." Nutrients 11(4).

Ortega, M. A., M. A. Alvarez-Mon, C. Garcia-Montero, O. Fraile-Martinez, J. Monserrat, L. Martinez-Rozas, R. Rodriguez-Jimenez, M. Alvarez-Mon and G. Lahera (2023). "Microbiota-gut-brain axis mechanisms in the complex network of bipolar disorders: potential clinical implications and translational opportunities." Mol Psychiatry 28(7): 2645-2673.

Rog, J., L. Lobejko, M. Hordejuk, W. Marciniak, R. Derkacz, A. Kiljanczyk, M. Matuszczak, J. Lubinski, M. Nesterowicz, M. Zendzian-Piotrowska, A. Zalewska, M. Maciejczyk and H. Karakula-Juchnowicz (2024). "Pro/antioxidant status and selenium, zinc and arsenic concentration in patients with bipolar disorder treated with lithium and valproic acid." Front Mol Neurosci 17: 1441575.

Sampath, V. P., S. V. Singh, I. Pelov, O. Tirosh, Y. Erel and D. Lichtstein (2022). "Chemical Element Profiling in the Sera and Brain of Bipolar Disorders Patients and Healthy Controls." Int J Mol Sci 23(22).

Shazia, Q., Z. H. Mohammad, T. Rahman and H. U. Shekhar (2012). "Correlation of oxidative stress with serum trace element levels and antioxidant enzyme status in Beta thalassemia major patients: a review of the literature." Anemia 2012: 270923.

Wolf, T. L., J. Kotun and J. H. Meador-Woodruff (2006). "Plasma copper, iron, ceruloplasmin and ferroxidase activity in schizophrenia." Schizophr Res 86(1-3): 167-171.

**Major points 5 and 6:** Similarly conclusions: "We observed inhibitory effect of titanium, manganese, and iodine on BD risk, on the contrary, magnesium, and nickel may increase the risk of BD." are not supported by any data in the work. How are Mg and Ni responsible for increasing the risk of BD, and how have the authors arrived at this conclusion just based on the analytical studies? The claim that "These findings will provide valuable insights into the potential influence of trace elements on the pathophysiology of BD and may have significant implications for prevention and treatment strategies. " is completely far fetched. There is no supporting evidence for these statements in the work carried out by authors.

**Authors’ response**:

Thanks for your suggestion. According to your advice, we have revised the abstract in the manuscript as outlined below: "*RCS results showed a non-linear correlation between the elements titanium, manganese, iodine, magnesium, nickel, zinc, strontium and adolescent BD. In addition, BKMR analysis showed a significant joint effect of multiple elements on adolescent BD when the concentrations of seven elements were at or above their 55th percentile compared with their median values.*" (see line 60 in the revised manuscript)

"*Our findings revealed that urinary titanium, manganese and iodine were negatively correlated with adolescent BD, while urinary magnesium and nickel were positively correlated with adolescent BD. These results provide some evidence of an association between* *urine trace elements and adolescent BD.*" (see line 64 in the revised manuscript)

In the results section, we have modified as below: "*We have observed the potential joint effect of Zn, Mn and I, and the interaction between Ni and Mn.*" (see line 319 in the revised manuscript)

In the discussion section, we have modified as below: "*BKMR analyses indicated a significant joint effect of mixture of seven elements (Ti, Mn, I, Mg, Ni, Zn, and Sr) on BD. The univariate exposure-response function revealed negative relationships between Ti, Mn, and I with BD, while urinary Mg, Ni, Zn, and Sr was positively associated with BD. Furthermore, the bivariate expose-response function shows that Zn, Mn and I have a potential correlation with adolescent BD. and Ni and Mn may also interact.*" (see line 350 in the revised manuscript)

In the conclusion section, we have modified as follows: "*Specifically, levels of Ti, Mn, and I were negatively correlated with BD in adolescents, while levels of Mg and Ni were positively correlated with BD. Furthermore, the combined effects of these seven elements (Ti, Mn, I, Mg, Ni, Zn and Sr) exhibited a significant correlation with BD.*" (see line 442 in the revised manuscript)

**Reviewer #3:** This case-control study investigates the association between various trace elements and the risk of bipolar disorder in adolescents.

The introduction clearly states the study's initial hypotheses and outlines its objectives. It also explains the statistical methods used to identify any potential relationships between urinary levels of multiple trace elements and bipolar disorder. Additionally, this section could benefit from further elaboration on the links between key elements studied and bipolar disorder, based on existing evidence. This would enhance readers' understanding and provide a better context for interpreting the study's results.

In the methods section, the study details the population under investigation, the timeframe and location of the research, the criteria for inclusion and exclusion, the questionnaires used, and the techniques employed to analyze the results.

The discussion section effectively compares the study's findings with those of similar research and is well-structured overall.

**Authors’ response**:

Thank you very much for your thoughtful and detailed review of our manuscript. We greatly appreciate your positive feedback on the study's introduction, methods, and discussion sections.

Once again, thank you for your comments and suggestions. We tried our best to improve the manuscript and made some changes marked in red in revised paper which will not influence the content and framework of the paper. We appreciate for Editors/Reviewers warm work earnestly and hope the correction will meet with approval.
